# Incentivizing the Public to Support Invasive Species Management: Eurasian Milfoil Reduces Lakefront Property Values

**DOI:** 10.1371/journal.pone.0110458

**Published:** 2014-10-15

**Authors:** Julian D. Olden, Mariana Tamayo

**Affiliations:** 1 School of Aquatic and Fishery Sciences, University of Washington, Seattle, Washington, United States of America; 2 Faculty of Life and Environmental Sciences, University of Iceland, Reykjavík, Iceland; Fudan University, China

## Abstract

Economic evaluations of invasive species are essential for providing comprehensive assessments of the benefits and costs of publicly-funded management activities, yet many previous investigations have focused narrowly on expenditures to control spread and infestation. We use hedonic modeling to evaluate the economic effects of Eurasian milfoil (*Myriophyllum spicatum*) invasions on lakefront property values of single-family homes in an urban-suburban landscape. Milfoil often forms dense canopies at the water surface, diminishing the value of ecosystem services (e.g., recreation, fishing) and necessitating expensive control and management efforts. We compare 1,258 lakeshore property sale transactions (1995–2006) in 17 lakes with milfoil and 24 un-invaded lakes in King County, Washington (USA). After accounting for structural (e.g., house size), locational (e.g., boat launch), and environmental characteristics (e.g., water clarity) of lakes, we found that milfoil has a significant negative effect on property sales price ($94,385 USD lower price), corresponding to a 19% decline in mean property values. The aggregate cost of milfoil invading one additional lake in the study area is, on average, $377,542 USD per year. Our study illustrates that invasive aquatic plants can significantly impact property values (and associated losses in property taxes that reduce local government revenue), justifying the need for management strategies that prevent and control invasions. We recommend coordinated efforts across Lake Management Districts to focus institutional support, funding, and outreach to prevent the introduction and spread of milfoil. This effort will limit opportunities for re-introduction from neighboring lakes and incentivize private landowners and natural resource agencies to commit time and funding to invasive species management.

## Introduction

Despite the long history of investigating the ecology of nonindigenous species [1], the scope of economic damages associated with species invasions has only recently received greater attention [2], [3]. Continental scale estimates suggest that thousands of invasive plants and animals have generated billions of dollars in economic losses [4]–[6]. These estimates, however, are conservative because they focus predominantly on expenditures to control the infestation and spread of invasive species. From an economic perspective, the full cost of biological invasions also includes the effects on host ecosystems and the human populations dependent on them [7]. The societal value that individuals give to both market (e.g., forestry) and nonmarket (e.g., landscape aesthetics) goods and services is also important to the economic valuation of damages incurred by invasive species. These values consider the market price of goods and services and people’s willingness to pay and sell them [8]–[10].

In freshwater environments, previous studies have largely focused on the economic impacts of invasive species on fisheries, power plants, water treatment facilities, and recreation [11], [12]. For example, the invasion of the rusty crayfish (*Orconectes rusticus*) into lakes in northern Wisconsin (USA) is estimated to generate damages of about $1.5 million USD annually to the panfish recreational fishery [13], and zebra mussels (*Dreissena polymorpha*) cost an estimated $267 million USD in lost power generation and drinking water treatment facilities in Lake St. Clair (USA) during the first 15 years of infestation [14]. However, a more complete understanding of the full spectrum of economic effects associated with aquatic invasive plants is needed to develop comprehensive policies and management strategies, as well as to incentivize the public to prevent future spread.

Eurasian milfoil (*Myriophyllum spicatum* L., herein referred to milfoil) is an ideal study organism to enhance our knowledge regarding the economic effects of aquatic invasive species because extensive information is available on the ecology and management of this invasive plant [15]. Native to Europe, Asia, and northern Africa, milfoil is now found on all continents except Australia and Antarctica, including almost all states and provinces of the United States and Canada [16]. This submersed perennial grows in a wide range of water temperatures, depths, and turbidities [15]. Milfoil can propagate through vegetative and sexual reproduction, although the former via stem fragments and runners provides the main mechanism of dispersal [17] by hitchhiking between waterbodies on trailered boats [18]. Milfoil invasions have become a major environmental nuisance in countless lakes across North American and globally, and many additional water bodies are susceptible to future invasions [19], [20].

Freshwater ecosystems are often severely impacted by milfoil invasion. Milfoil form dense canopies in the water column (extending to the water surface) altering water chemistry, displacing native plants, and creating habitats that are unsuitable for wildlife [15], [21], [22]. The costs of controlling milfoil, which include mechanical harvesting, underwater cultivation, diver hand-pulling, water level manipulation, biological control, and aquatic herbicide application, exceed many millions USD annually [23]. For example, during a 15 year period (1985–2010) over $5 million USD was spent to control milfoil in Lake George (New York, USA) [24]. Moreover, milfoil can diminish the value of services like recreation, by hindering boating and swimming activities. In the Truckee River watershed (California and Nevada, USA), estimates of a potential decline in recreation values of only 1% due to the spread of milfoil were at least $500,000 USD annually [23]. Milfoil can also impact provisioning services such as agriculture and electricity generation, by reducing water circulation in irrigation projects and blocking water intakes in power plants.

In this study we evaluate the potential economic impacts of aquatic invasive plants on lakefront properties, using Eurasian milfoil as an illustrative example. Such impacts are largely unexplored (but see [25], [26]), yet are critical to determine the benefits and costs of different strategies to manage invasive aquatic plants and to actively engage the public into management actions regarding the spread of non-native species. Specifically, we evaluate the economic effects of milfoil invasions on lakefront property values of single-family homes in a region of western Washington (USA) by applying a hedonic modeling framework. Furthermore, we assess the welfare effect of milfoil invading one additional lake in our study area in order to inform future prevention efforts.

## Methods

### Study region

**Figure 1:**
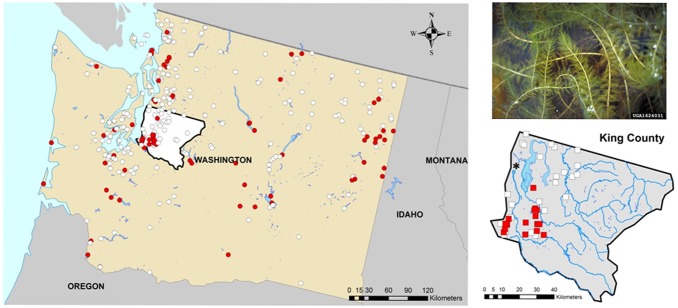
Location of milfoil presences (red filled circle) and absences (white empty circles) in lakes of Washington, USA, including King County (bottom right) containing 17 invaded lakes (filled squares) and 24 uninvaded lakes (empty squares). The city of Seattle, Washington (2010 population size of 608,660) is indicated as *.

Our study focused on lakefront properties in Pacific Northwest region of North America, specifically King County, Washington (USA). This county has the highest population density in the state (1,931,249 residents according to the 2010 census) and it is intersected (north-south) by the Interstate-5 highway, which serves as an invasion corridor for non-native plant species both in terms of high human populations (introduction via the aquarium trade) and movement of recreational boaters (introduction via entrainment on trailer boats). Lakes throughout King County are located along a distinct urban-rural land use gradient, and many have primary residences and support public recreation [27], making our study distinct from previous investigations examining milfoil impacts in rural landscapes. We assessed the economic effect of milfoil by comparing 1,258 lakeshore property sale transactions of single family homes in 41 small lakes (lake area <1 km^2^) from 1995 to 2006 (Figure 1), prior to the 2007 decline of housing prices in the county and the state [28]. Although the county has >150 small lakes, we were limited to those containing complete datasets for sales transactions, structural, locational, and environmental characteristics (see below). The dataset consisted of 17 lakes with milfoil during the study period (611 total transactions) and 24 un-invaded lakes (647 total transactions) located in a predominantly urban-suburban landscape. The exact date when milfoil invaded each lake is unknown; however, based on county records [29] and personal communication with county officials 15 of the lakes were invaded prior to 1995 and two prior to 1999. Because the invasion dates of the latter two lakes were unclear, we treated them as being invaded throughout the 12-year study period. Unfortunately data on milfoil density is lacking for many lakes, therefore our analysis focused on presence/absence. Data sources were the King County Department of Assessments, King County Department of Natural Resources and Parks, and the Washington Department of Ecology.

### Statistical approach

We used hedonic modeling to quantify the effect of milfoil invasions on lake property values. This technique has proven useful in estimating the economic value of nonmarket amenities, for example, the effect of water quality on the recreational and aesthetic value of freshwater resources and shoreline properties [30]–[32]. We provide a brief description of this approach below, but refer the reader to Rosen [33] for further information. Hedonic modeling partitions a composite good (e.g., property value) into its defining characteristics and estimates the value (i.e., implicit price) of each characteristic. The relationship between the market price of the good and its attributes is the hedonic price function. We followed Halstead et al. [34] by defining the hedonic price function as *HP = f(S, L, E)*, where *HP* represents home (property) price, *S* are structural characteristics (e.g., house size), *L* are locational characteristics (e.g., parcel density), and *E* are environmental characteristics (e.g., water clarity).

We modeled *HP* as a function of key property characteristics (*S*, *L*, *E*) to generate the value (i.e., marginal implicit price) consumers give to each characteristic. These estimated values were then used to evaluate the effect of milfoil presence on property value. A suite of independent variables (Table 1) that previous studies have identified as important in determining lakeshore property prices were analyzed [25], [35]. We modeled *HP* as a linear function of these variables for ease of interpretation and because this functional form has been used extensively in hedonic analyses [36], [37]. Given that properties around a lake are influenced by the same lake-specific characteristics, we considered each lake a cluster of property sales and characteristics (see [25]). Unobserved lake characteristics can lead to endogeneity, whereby an independent variable is correlated with the error terms in the model, resulting in biased estimates of model coefficients. We used two-stage least squares regression to account for correlations between the error terms of the dependent variable and the independent variables. This regression uses instrumental variables that are uncorrelated with the error terms but are correlated to the endogenous variables to estimate the values of the endogenous variables (first stage), and then uses these estimated values to model the dependent variable (second stage) (see [38]).

**Table 1:** Structural, locational, and environmental independent variables used in the hedonic analysis of property sales price (*).

Variable	Description	Mean	S.E.
Sales price*	Selling price of the property (land + house; 2006 USD)	502312.8	23942.4
*Structural*			
Lot size	Size of a parcel (m^2^)	2394.5	216.2
Frontage	Shoreline frontage of a property (m^2^)	22.0	1.1
House size	Total living area (m^2^)	204.8	6.6
House age	Age of a house (years)	39.5	1.6
*Locational*			
Boat launch	Presence of a public boat launch	0.6	0.1
Fish stocking	Presence of fish stocking for recreational angling	0.7	0.1
Parcel density	Number of parcels per km^2^	512.1	35.6
*Environmental*			
Milfoil presence	Presence of Eurasian milfoil	0.4	0.1
Lake area	Surface area of a lake adjacent to the property (km^2^)	0.2	0.03
Temperature	Mean surface water temperature during the milfoilsummer growing season (°C)	19.8	0.3
Water clarity	Mean Secchi depth of the lake during the milfoilgrowing season (m)	3.4	0.2

### Hedonic model structure

The hedonic model comprised of lakefront property sales price as the dependent variable, which was deflated to 2006 property values (USD) using the house price index (purchase only) from the US Federal Housing Finance Agency. Independent variables used in the analysis included structural characteristics (i.e., house size, house age, lot size, frontage), locational attributes (i.e., presence of a public boat launch, recreational fish stocking, lakefront parcel density) and environmental descriptors (i.e., presence of Eurasian milfoil, lake area, water temperature, water clarity) (Table 1).

The choice of endogenous and instrumental variables is influenced by geography and the specific characteristics of the focal property market (e.g., [25], [26]). Our variable selection and model structure reflects a property market composed of primary residences in an urban-suburban landscape. Below we describe the endogenous and instrumental variables used in the hedonic model (independent variables are listed Table 1), noting that endogenous variables refer to factors whose values are determined by the state of other variables in the system and instrumental variables are hypothesized to be correlated to the endogenous variables but not to the dependent variable (property sales price).

Milfoil presence was treated as an endogenous variable; a choice supported by Horsch and Lewis [25] who showed the endogeneity of milfoil presence in a hedonic model. Recreational boaters commonly spread milfoil among lakes [18] and lake characteristics that increase the desirability for recreation are also attractive for homeownership. However, it is difficult to quantify many of these desirable characteristics, thus increasing the likelihood that milfoil presence is endogenous [25]. In our housing market, we used the occurrence of a public boat launch and fish stocking as instrumental variables because they are linked to recreational boating (i.e., the primary vector of milfoil introduction into lakes). Our choice is supported by the fact that all properties have direct dock assess to the lake and self-sustaining recreational fish populations exist in those lakes that are not stocked; therefore, these factors likely have little effect on a homeowner’s willingness to pay in our housing market. We also used water clarity, lake area, and water temperature as instrumental variables for milfoil presence due to their influence on habitat suitability for milfoil establishment [15], [20], [27]. Although water quality is known to have an effect on property values of housing markets (e.g., [30], [32], [39]), this effect is unlikely to be manifested our property market where >90% of the study lakes had water clarity >2 m, with an average of 3.4 m and little variability (SD = 0.19 m). Given this, differences in water clarity are likely imperceptible to potential property buyers. Lake area and water temperature were also very similar among our lakes given their similar glacial history and elevation. Taken together, our housing market is characterized by similar sized lakes with good water clarity and similar water temperatures; therefore, it is unlikely that these attributes significantly affected a homeowner’s willingness to pay.

A series of regression models were developed and compared using the modified Akaike’s Information Criterion for small samples (AICc). AICc is a model selection technique based on the trade-off between model accuracy and parsimony [40]. Akaike weights were calculated with the AICc values to determine the relative likelihood that each model is the best model given the data and the other candidate models. Statistical analyses where conducted using PASW 18 (IBM SPSS).

We estimated the aggregate cost of milfoil invading one additional small lake in our study area by discounting a homeowner’s willingness to pay for a property on a lake free of milfoil by 5% (same rate as in [25]) to estimate the average annual marginal willingness to pay, and then multiplying this annual average with the mean number of parcels for our study lakes (n = 80 parcels).

## Results

The presence of Eurasian milfoil had a significant negative effect on property values; mean reduction in property values was $94,385 USD, ranging from −$92,558 to −$94,670 USD according to the top three competitive models (Table 2). Based on an average sale price of $502,313 across all study lakes, the negative effect of milfoil presence corresponds to a 19% decline in mean property values.

**Table 2:** Hedonic analysis results for the two-stage least squares regression model predicting property price as a function of key independent variables describing structure, location, and the environment (see Table 1).

	Model 1			Model 2			Model 3		
Variable	Coefficient	Sig.	S.E.	Coefficient	Sig.	S.E.	Coefficient	Sig.	S.E.
Constant	−63891.9		97303.1	−29052.8		117533.5	−95790.7		149910.1
Milfoil presence	−94385.4	**	46712.9	−94670.0	*	49174.7	−92558.0	*	48255.6
House size	2608.1	***	344.2	2474.0	***	703.9	2681.6	***	726.0
Lot size				−1.2		13.2	3.1		12.9
Parcel density	102.8		70.3				115.4		78.6
Lake area	209407.8	**	81848.0	154409.5	*	74073.6	215595.0	**	84521.5
Water clarity	−7430.3		11160.2	−6962.7		12820.2	−6954.9		12600.7
AICc	23.931			23.986			23.998		
Relative likelihood	1.000			0.969			0.963		

See text for discussion of the endogenous variable (milfoil presence) and instrumental variables. Reported are the top three candidate models according to Akaike’s Information Criterion for small samples (AICc) with their associated parameter coefficients and standard errors. The relative likelihood that the model is the best model given the data is denoted.

Significant levels: *P<0.10, **P<0.05, ***P<0.01.

The hedonic analysis revealed that larger homes located on lakes with larger surface areas had a significant positive effect on property values, on average selling for $2,600 per m^2^ and $209,400 per km^2^ more, respectively (Table 2). Parcel density showed a negligible effect on property value, whereas water clarity negatively influenced property sales prices, though not statistically significant. All model parameters, except for water clarity and parcel density, reflected the anticipated directional effect on property value (Table 1).

A homeowner’s marginal willingness to pay for a waterfront property on a lake free of milfoil was on average $94,385 (model 1 in Table 2), resulting in an average annual marginal willingness to pay of $4,719 (using a 5% discount rate). The aggregate cost of milfoil invading one additional study lake was $377,542 per year ($4,719×80 lakefront parcels).

## Discussion

A broader understanding of the economic impacts of aquatic invasive plants is essential for promoting changes in policy and engaging more diverse stakeholder participation, such as lakefront property owners and recreational boaters, in the management of natural resources. Only until the full cost of biological invasions is considered (i.e. beyond control expenditures), will the optimal economical management of invasive species be possible [41]. Our study demonstrates that aquatic invasive plants can have dramatic economic impacts on the sale value of lakefront properties. The presence of milfoil in a lake results in an “invisible tax” on the real estate market by substantially reducing property values an average of over $94 thousand USD, translating to 19% decline in value. We note that our estimates did not consider the level of infestation, the implementation of management actions, nor the losses to recreation.

Similar economic damages have been reported in northern Wisconsin, where waterfront property values in a popular recreational and rural area declined by approximately 8% after milfoil invaded a lake [25]. Furthermore, the process of milfoil infestation in five Vermont lakes (USA) resulted in property values that decreased by <1% to 16% depending on the level of infestation [26]. Both these studies examined rural properties containing mostly vacation homes (secondary residences) located in forested landscapes; our study adds to this understanding by demonstrating economic impacts to property values of primary residences in urban settings. Taken together, the negative effect of milfoil on property values of primary and secondary homes in different regions and landscape settings, suggests that the economic impacts of aquatic invasive plants are widespread and may be greater in urbanized landscapes. We recognize that milfoil presence/absence may overestimate economic impacts compared to plant density [26]. Additional studies that include detailed estimates of milfoil infestation (abundance) at the time of purchase, distance of property to nearest milfoil colony, and the level of property buyer knowledge of milfoil are warranted [34]. By contrast, the economic impacts of milfoil may be undervalued if those properties on highly infested lakes are the most difficult to sell and therefore remain on the market.

The costs of preventing new invasions of aquatic weeds are often thought to be greater than the benefits, thus leading to inaction. Our study, however, indicates there are benefits to preventing the spread of milfoil given that the invasion of one additional study lake leads to a high aggregate cost of over $375 thousand USD annually. This aggregate cost represents a third of the amount spent annually ($1 million USD) on managing milfoil across Washington State [42]. The knowledge that an invasion of milfoil can lead to a significant decline in property values provides the public an economic incentive to invest in prevention and/or control strategies [43]. Moreover, reductions in property values also translate directly to substantially losses in property taxes garnered by local governments. Thus the economic impacts of milfoil invasions may extend well beyond the infested lakefront properties by reducing local government revenue.

Lakefront property owners stand to benefit greatly (higher property values) from preventing milfoil invading their lake. In addition, it is necessary to engage recreational boaters in prevention efforts as well regardless whether or not they live on a lake, because they are an important dispersal vector of milfoil and other aquatic invasive species [18], [19], [44]. When recreational boaters spread milfoil into a new lake they are inadvertently creating hidden costs (negative externalities) to other lake users of the newly invaded lake; these costs include lower property values, reduction of biodiversity, and diminished recreational experience, among others.

Property owners could also benefit from aquatic weed control. Zhang and Boyle [26] showed that control efforts on a heavily infested lake that reduced milfoil areal coverage from 81–100% to 61–80% could offset losses to property values caused by the invasion. Similarly, we expect that properties on lakes where milfoil densities have been reduced will likely experience a reduced negative price effect. We did not consider milfoil management effects in our analysis because it requires a treatment to have taken place before the property transaction but within the same year. If the treatment were to take place after the transaction, the associated benefit to a selling property would not yet be capitalized into property price; ignoring expectations or knowledge of a pending treatment.

A key component for long-term management of invasive species is the participation of multiple groups representing ecological and socio-economic perspectives [45], [46]. Often, however, engaging stakeholder groups is difficult because each entity may have different attitudes towards invasive species and resource allocation [47], [48]. For example, a study of stakeholder perceptions about invasive species in the Doñana wetland (Spain), revealed remarkably different viewpoints among parties, which included local users, tourists, and conservation professionals [49]. People were more willing to support and pay for management of invasive species (including eradication) when they had a higher level of education, and a better understanding of the study. Therefore, to successfully manage Eurasian milfoil and other invasive species it is important to embrace the diversity of perceptions held by the stakeholders, by employing strategies (e.g., involving stakeholders at the beginning of the decision-making process) that promote cooperative participation and communication among parties [46], [49].

Economic research on invasive species is essential for comprehensive assessments of the benefits and costs of management strategies aimed at increasing the effectiveness of publicly funded programs [50], [51]. Prevention of future introductions and control of existing invasions are powerful management options [52], however, the ecological and economic benefits of these actions must be better illustrated. Individual costs of milfoil invasions (this study; [25], [26]) coupled with local, regional and national costs associated with lost recreation, agriculture and power generation (e.g., [23]) make for a compelling case that even modest expenditures on prevention could help avoid substantial economic impacts and help preserve freshwater ecosystems. Public-derived funding for aquatic weed management in the United States is generally provided through state-derived sources and the creation of Lake Management Districts that allow lake property owners to tax themselves and other lake users to collect funds for various prevention and control activities. Only three of our study lakes were represented by a Lake Management District at the time of our analysis. We recommend coordinated efforts across the management mosaic (*sensu* [41]), whereby networks of Lake Management Districts operate together to focus institutional support, funding, and outreach to prevent the introduction and spread of milfoil. This effort will limit opportunities for re-introduction from neighboring lakes and incentivize homeowners to commit time and funding to invasive species management, including the education of transient boaters.
